# Hypoxic microenvironment as a crucial factor triggering events leading to rupture of intracranial aneurysm

**DOI:** 10.1038/s41598-023-32001-z

**Published:** 2023-04-04

**Authors:** Isao Ono, Tomomichi Kayahara, Akitsugu Kawashima, Akihiro Okada, Susumu Miyamoto, Hiroharu Kataoka, Hiroki Kurita, Akira Ishii, Tomohiro Aoki

**Affiliations:** 1 Department of Molecular Pharmacology, Research Institute, National Cerebral and Cardiovascular Center, 6-1 Kishibe-Shinmachi, Suita, Osaka 564-8565 Japan; 2 Core Research for Evolutional Science and Technology (CREST) from Japan Agency for Medical Research and Development (AMED), National Cerebral and Cardiovascular Center, Osaka, Japan; 3 Department of Neurosurgery, Kyoto University Graduate School of Medicine, Kyoto, Japan; 4 Department of Cerebrovascular Surgery, Saitama Medical University International Medical Center, Saitama, Japan; 5 Department of Neurosurgery, Tokyo Women’s Medical University Yachiyo Medical Center, Chiba, Japan; 6 Department of Neurosurgery, National Cerebral and Cardiovascular Center, Osaka, Japan

**Keywords:** Molecular biology, Cardiology, Diseases, Medical research

## Abstract

Subarachnoid hemorrhage being the rupture of intracranial aneurysm (IA) as a major cause has quite poor prognosis, despite the modern technical advances. Thereby, the mechanisms underlying the rupture of lesions should be clarified. Recently, we and others have clarified the formation of vasa vasorum in IA lesions presumably for inflammatory cells to infiltrate in lesions as the potential histopathological alternation leading to rupture. In the present study, we clarified the origin of vasa vasorum as arteries located at the brain surface using 3D-immunohistochemistry with tissue transparency. Using Hypoxyprobe, we then found the presence of hypoxic microenvironment mainly at the adventitia of intracranial arteries where IA is formed. In addition, the production of vascular endothelial growth factor (VEGF) from cultured macrophages in such a hypoxic condition was identified. Furthermore, we found the accumulation of VEGF both in rupture-prone IA lesions induced in a rat model and human unruptured IA lesions. Finally, the VEGF-dependent induction of neovessels from arteries on brain surface was confirmed. The findings from the present study have revealed the potential role of hypoxic microenvironment and hypoxia-induced VEGF production as a machinery triggering rupture of IAs via providing root for inflammatory cells in lesions to exacerbate inflammation.

## Introduction

Intracranial aneurysm (IA), a regional bulging of intracranial arteries, can develop subarachnoid hemorrhage (SAH) after rupture and becomes a major cause of it^1^. Nowadays, more and more cases with unruptured IAs are found in clinical settings, meaning the presence of a chance to apply medical interventions to prevent rupture. Because of poor outcome once after the onset of SAH in spite of the technical advancement of the treatment and the medical care^1,2^, the development of a novel therapeutic strategy to prevent rupture of IA lesions is mandatory for public health. For this purpose, precise molecular mechanisms underlying the process leading to rupture of the lesions should be clarified to identify therapeutic targets.

Through a series of studies mainly using animal models of IAs, we and others have successfully defined IA as a chronic inflammatory disease affecting intracranial arteries mainly at bifurcation sites^3–17^. Here, we have recently proposed the potential mechanisms; the formation of vasa vasorum provides the root for inflammatory cells, mainly macrophages and neutrophils, to facilitate the infiltration in lesions and triggers the exacerbation of inflammation in situ leading to rupture of IA lesions^10,18^. In addition, the contribution of neutrophils to the onset of SAH is also supported by the experiment in which GM-CSF, Granulocyte Macrophage colony-stimulating Factor, facilitated rupture of IAs via the increase in infiltrating neutrophils^10^. Thereby, the underlying machinery to induce vasa vasorum at the adventitia could be a therapeutic target to prevent rupture of IAs. Here, hypoxia and the hypoxia-induced expression of angiogenic factor, most typically vascular endothelial growth factor (VEGF), are well-established and potent inducer of angiogenesis^19–21^, the formation of vasa vasorum in case of IAs^18^. Because intracranial arteries histologically lack vasa vasorum^22^, the adventitia of intracranial arteries consists of the hypoxic microenvironment in nature and the past reports have indeed demonstrated the oxygen partial pressure in the adventitia as approximate 40 mmHg^23–25^. We therefore examined the presence of hypoxia in rupture-prone or ruptured lesions and the expression of angiogenic factors in lesions.

## Methods

### IA models of rats and histological analysis of induced IA

10-week-old female Sprague − Dawley rats were purchased from Japan SLC (Slc:SD, n = 20, Shizuoka, Japan). Animals were maintained on a light/dark cycle of 12 h/12 h, and had a free access to chow and water. To induce IAs^10,18,26^, rats were subjected to the bilateral ovariectomy, the ligation of the left carotid artery, the right external carotid artery and the right pterygopalatine artery, and systemic hypertension achieved by the combination of a high salt diet and the ligation of the left renal artery under general anesthesia by the inhalation of Isoflurane (induction; 5.0%, maintenance; 1.5–2.0%, #IYESC-0001, Pfizer Inc., New York, NY). Immediately after surgical manipulations, animals were fed the chow containing 8% sodium chloride and 0.12% 3-aminopropionitrile (#A0408, Tokyo Chemical Industry, Tokyo, Japan), an irreversible inhibitor of Lysyl Oxidase catalyzing the cross-linking of collagen and elastin. At 16 weeks after above surgical manipulations, animals were deeply anesthetized by the inhalation of Isoflurane (5.0%, #IYESC-0001, Pfizer Inc.), and transcardially perfused with 4% paraformaldehyde solution. The circle of Willis was then stripped from brain surface and an IA lesion induced at an anterior communicating artery or a posterior communicating artery was dissected.

### Tissue transparency and immunohistochemistry

Tissue transparency was done using paraformaldehyde-fixed specimens and CUBIC-L and CUBIC-R solutions (#T3740 or #T3741, TCI chemicals, Tokyo, Japan) as manufacturer’s instructions. Immunohistochemistry to visualize medial smooth muscle cells was done using a CUBIC-HV 3D Immunostaining Kit and mouse monoclonal anti-α-smooth muscle actin antibody conjugated with Cy3 (#C6198, clone 1A4, Sigma, St. Louis, MI) as manufacturer’s instructions. The images were acquired by a confocal laser microscopy (FV3000, Olympus, Tokyo, Japan).

### Immunohistochemistry

In specimens from animals, 5-µm-thick frozen sections were prepared from dissected IA lesions or anterior cerebral—olfactory artery bifurcations, aorta or liver prepared as described above. After blocking with 3% donkey serum (#AB_2337258, Jackson ImmunoResearch), slices were incubated with primary antibodies followed by incubation with secondary antibodies conjugated with a fluorescence dye (Jackson ImmunoResearch). In some experiments, the primary antibody conjugated with a fluorescence dye was used. Finally, fluorescent images were acquired on a confocal fluorescence microscope system (FV3000, Olympus, Tokyo, Japan).

In specimens from human cases, dissected human specimen was fixed in formalin solution and embedded in paraffin. 4-µm-thick slices were then prepared for immunohistochemical analysis. After deparaffinization and blocking with 10% donkey serum (Jackson ImmunoResearch), slices were incubated with primary antibodies followed by incubation with secondary antibodies conjugated with fluorescence dye (Jackson ImmunoResearch). Finally, fluorescent images were acquired on a confocal fluorescence microscope system (FV3000, Olympus).

The antibodies used were as follows; mouse monoclonal anti-α-smooth muscle actin antibody (#M0851, Dako, Agilent, Santa Clara, CA), mouse monoclonal anti-α-smooth muscle actin antibody conjugated with Cy3 (#C6198, Sigma), mouse monoclonal anti-CD68 antibody conjugated with Alexa Fluor 647 (#sc-20060AF647, Santa Cruz Biotechnology, Dallas, TX), mouse monoclonal anti-VEGF antibody (clone VG1, #NB100-664, Novus Biologicals, LLC, Centennial, CO), mouse monoclonal anti-Pimonidazole antibody (clone MAb1, #HP1-100, Hypoxyprobe, Inc., Burlington, MA), Alexa Fluor 488-conjugated donkey anti-mouse IgG H&L antibody (#A21202, Thermo Fisher Scientific, Waltham, MA), Alexa Fluor 594-conjugated donkey anti-mouse IgG H&L antibody (#A21203, Thermo Fisher Scientific).

### Detection of hypoxic cells and tissues in vivo

To detect hypoxic cells or tissues in vivo, Hypoxyprobe was used (Hypoxyprobe, Inc.). This probe is the derivative of Pimonidazole which binds to hypoxic cells and can be detected in immunohistochemistry using the specific antibody for Hypoxyprobe delivered from the company. 60 mg/kg of Hypoxyprobe was intraperitoneally injected in rats and after 165 min the specimens were harvested subjecting to immunohistochemistry as in the above section.

### Induction of vasa vasorum from arteries at brain surface by VEGF

Burr hole was prepared in the skull of rats by high-speed drill system under general anesthesia as described above and the sheets for the slow-release of VEGF was placed on the brain surface (MedGel II from Nitta-gelatin (♯PI9, Osaka Japan) and recombinant human VEGF (1 μg/head, #564-RV-010/CF, lot #CWC0620081) from R&D systems, Inc. (Minneapolis, MN) resolved in phosphate buffered saline containing 0.1% human serum albumin (#A1653-5G, lot #SLBG2676V, Sigma)). The induction of vasa vasorum was then assessed by histological examination and by immunohistochemistry on 6th day as described above.

### Cell culture and loading hypoxia on cultured cells

RAW264.7 cell line, NIH3T3 cell line and U937 cell line were purchased from ATCC (#TIB-71 for RAW264.7, #CRL-1658 for NIH3T3, #CRL-1593.2 for U937, Manassas, VA). Cells were maintained in Dulbecco's Modified Eagle Medium (#044-29765, Fujifilm Wako Chemicals, Osaka Japan) supplemented with 10% fetal bovine serum (#FB-1365/500, lot#11953, biosera, Nuaille, France) (RAW264.7 cells and NIH3T3 cells) or RPMI-1640 (#189-02025, Fujifilm Wako Chemicals) with 10% fetal bovine serum (#FB-1365/500, lot#11953, biosera) (U937 cells).

To load hypoxia, cells were incubated in a hypoxia chamber system (0.5% or 5%, #Bionix-2, Sugiyama-Gen Co., Ltd., Tokyo, Japan) for 8 h.

### RNA purification and quantitative real time (RT)-PCR analysis

Total RNA was purified from treated cells and reverse-transcribed using a RNeasy Mini Kit (#74106, QIAGEN, Hilden, Germany) and a High-capacity cDNA Reverse Transcription Kit (#4368813, Life Technologies Corporation, Carlsbad, CA), according to the manufacturers’ instructions. For quantification of gene expression, quantitative RT-PCR was performed on a LightCycler 480 (Roche, Indianapolis, IN) with a TB Green Premix Ex Taq II (#RR820, TAKARA BIO INC., Shiga, Japan) and primer sets specific for each gene of interests (Supplementary Table S1). Expression of *Actb* (a gene coding β-actin) was used as internal controls. For quantitation, the second derivative maximum method was used for determining the crossing point.

### Concentration of VEGF in the supernatant from hypoxia-loaded RAW264.7 cells

The supernatant from hypoxia-loaded RAW264.7 (0.5%, 0–24 h) was collected subjecting to ELISA (#RSD-MMV00-1, R&D Systems) to examine the concentration of VEGF according to the manufacture’s indications.

### Statistics

Data are shown as Mean ± S.E.M. Differences between the two groups were examined using a non-parametric Mann–Whitney test. Differences more than three groups were examined using a Kruskal–Wallis test followed by a Steel test. A p value smaller than 0.05 was defined as statistically significant.

### Ethics approval and consent to participate

Human unruptured IA samples and control arterial walls (superficial temporal artery) were obtained during neck clipping with the written informed consent. The use of human samples in the present study was approved by the Ethics Committee at Kyoto University Graduate School and Faculty of Medicine (Approval Number #E2540 and #R0601), National Cerebral and Cardiovascular Center (Approval Number #M29-050, #R21012), and Tokyo Women’s Medical University Yachiyo Medical Center (Approval Number #4106) where samples were prepared. Also the written informed consent was obtained from each case. All of the following experiments, including animal care and use, complied with the National Institute of Health’s Guide for the Care and Use of Laboratory Animals and complied with the National Institute of Health’s Guide for the Care and Use of Laboratory Animals and were approved by the Institutional Animal Care and Use Committee of National Cerebral and Cardiovascular Center (Approval Number; #20003, #21015, #22041). The present manuscript adheres to the ARRIVE (Animal Research: Reporting of In Vivo Experiments) guidelines for reporting animal experiments.

## Results

### The identification of the origin of vasa vasorum as arteries at brain surface

**Figure 1:**
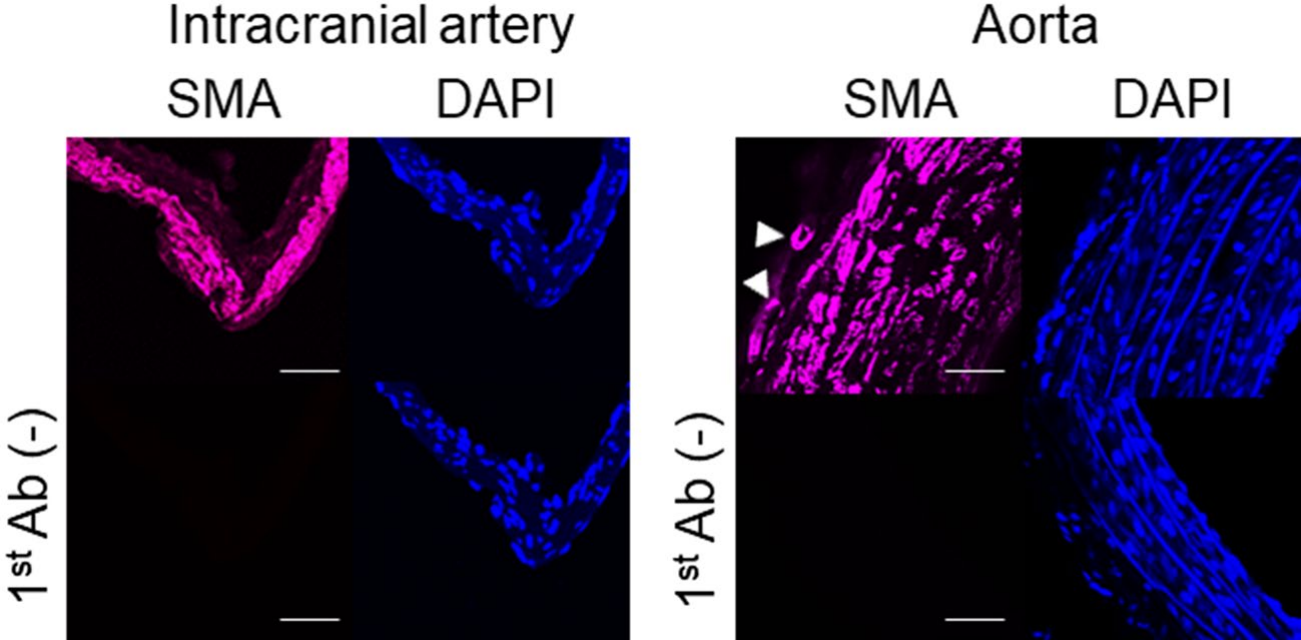
The absence of vasa vasorum in intracranial arteries. The images of immunofluorescent staining of intracranial arteries (left panels) or aorta (right panels) for the marker for smooth muscle cell, α-smooth muscle actin (SMA, red) and nuclear staining by DAPI (blue) are shown. Arrowheads indicate the vasa vasorum at the adventitia. Scale bar: 50 μm.

**Figure 2:**
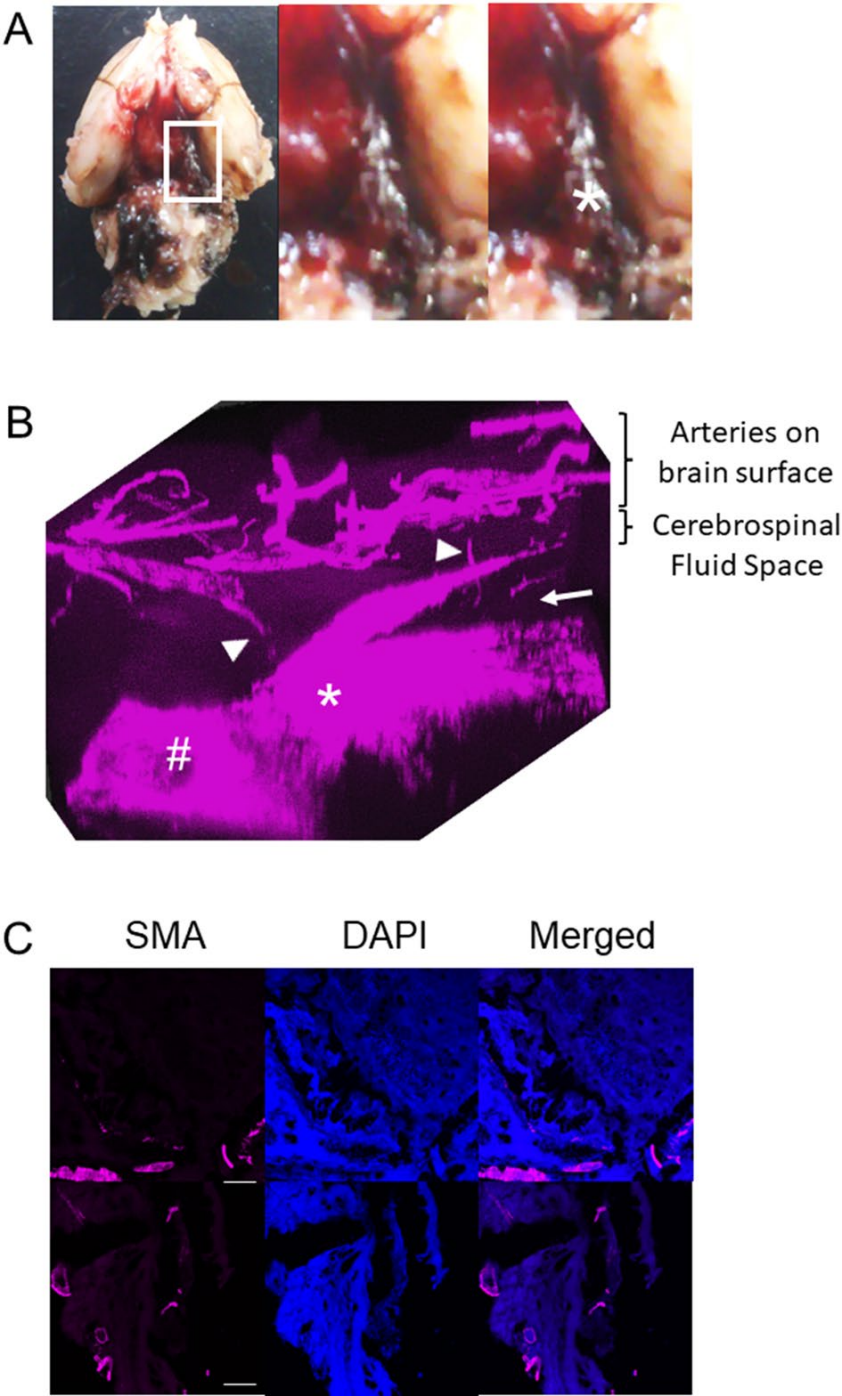
The origin of vasa vasorum migrating into the adventitia of intracranial aneurysmal lesions. Vasa vasorum running from arteries on brain surface. The macroscopic image of the brain before tissue transparency is shown in (**A**). The magnified images corresponding to the white square in the left panel are shown on the right. White asterisk indicates the ruptured intracranial aneurysm (IA). IA lesions and the surrounding structure were visualized by immunohistochemistry for medial smooth muscle cells with tissue transparency. The images of immunofluorescent staining of IA lesions for the marker for smooth muscle cell, α-smooth muscle actin [SMA, red in (**B,C**)], nuclear staining by DAPI [blue in (**C**)], and merged images (**C**) are shown. The images in (**C**) are from slices to confirm the presence of vasa vasorum in IA lesions. Scale bar: 50 μm. Arrowheads; arteries from the brain surface, Arrow; the point of rupture, asterisk; dome of the IA, hash; the parent artery.

Because intracranial arteries histologically lack vasa vasorum^22^ (Fig. 1), we examined where vasa vasorum came from by the combined technique of immunohistochemistry to visualize vascular smooth muscle cells with tissue transparency. In ruptured IA walls, arteries located on brain surface run toward the dome of IAs especially around the site of rupture where signals for the marker for vascular smooth muscle cells, α-smooth muscle actin, were absent (Fig. 2A,B). The presence of vasa vasorum with medial smooth muscle cells around the site of rupture in IAs was also confirmed in slices (Fig. 2C).

### The presence of hypoxic microenvironment at the adventitia of intracranial arteries

**Figure 3:**
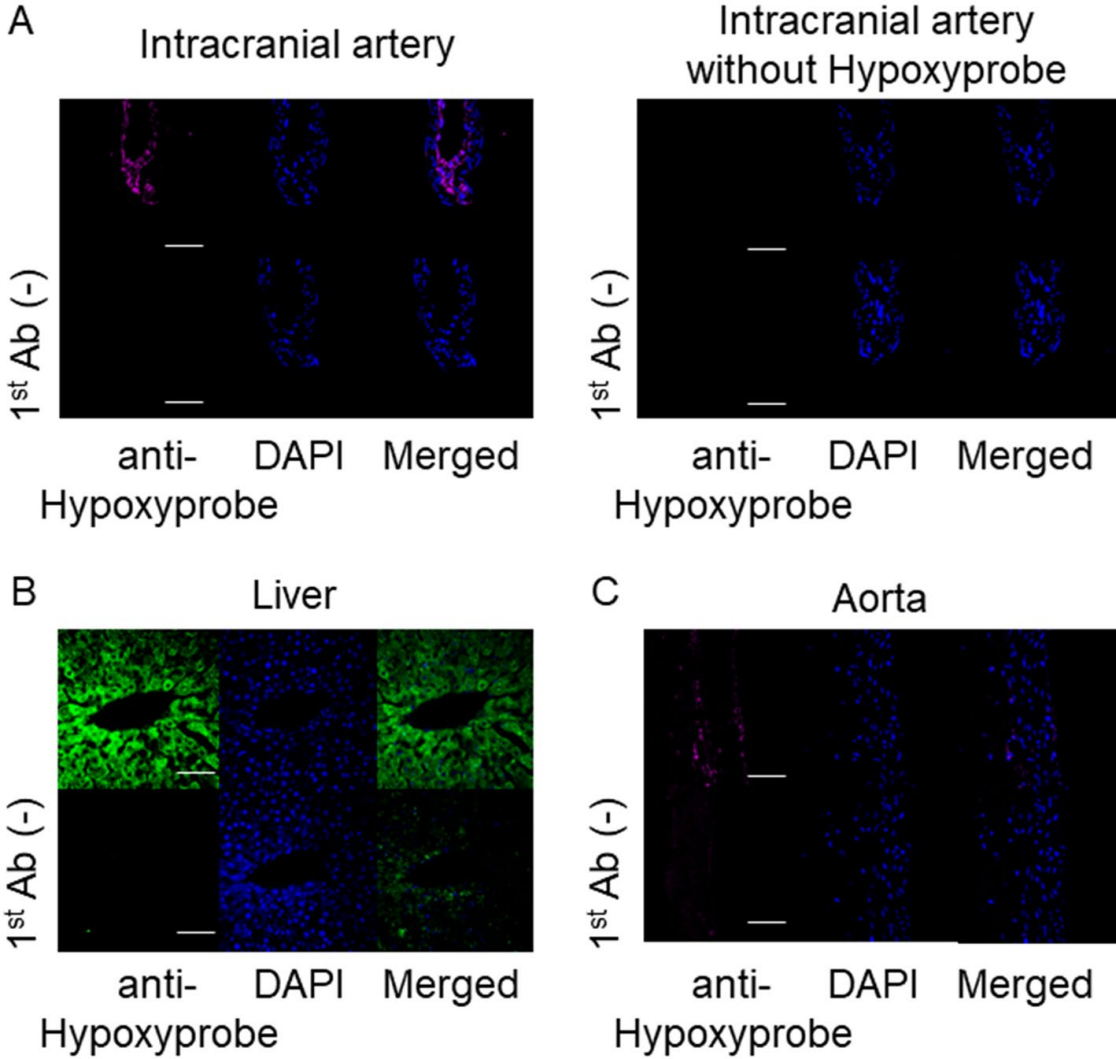
Presence of hypoxic microenvironment in the adventitia of intracranial arterial walls. Hypoxyprobe, the derivative of Pimonidazole, was administered to rats (60 mg/kg) to label hypoxic cells or tissues and the presence of this probe in intracranial arterial walls was detected in immunohistochemistry using the specific antibody. The images of immunofluorescent staining of intracranial arterial walls (**A**), liver served as a positive control (**B**) or aorta (**C**) for hypoxyprobe [red in **(A,C**), green in (**B**)], nuclear staining by DAPI (blue), and merged images are shown. The images from immunohistochemistry without a primary antibody or ones from specimens without the administration of hypoxyprobe were served as a negative control. Scale bar: 50 μm.

We first hypothesized that the adventitia of intracranial arteries was under the hypoxic condition because of the lack of vasa vasorum^22^ and the low oxygen partial pressure in CSF^23–25^. To assess the hypothesis, Hypoxyprobe, the derivative of Pimonidazole which binds to hypoxic cells, was administered to rats subjecting to the immunohistochemistry using the specific antibody to examine the presence of this probe in intracranial arterial walls, liver used as a positive control^27^or aorta. As a result, the signals for Hypoxyprobe were observed in cells located at the adventitia of intracranial arterial walls (Fig. 3A) and the cells located around the central vein in liver served as a positive control (Fig. 3B), supporting above hypothesis. Intriguingly, the positive signals for Hypoxyprobe could be only sparsely observed in aorta in which vasa vasorum was present (Fig. 3C), indicating the originality of intracranial arterial walls.

### Induction of VEGF and other angiogenic factors in cultured macrophages or fibroblasts under hypoxic condition

**Figure 4:**
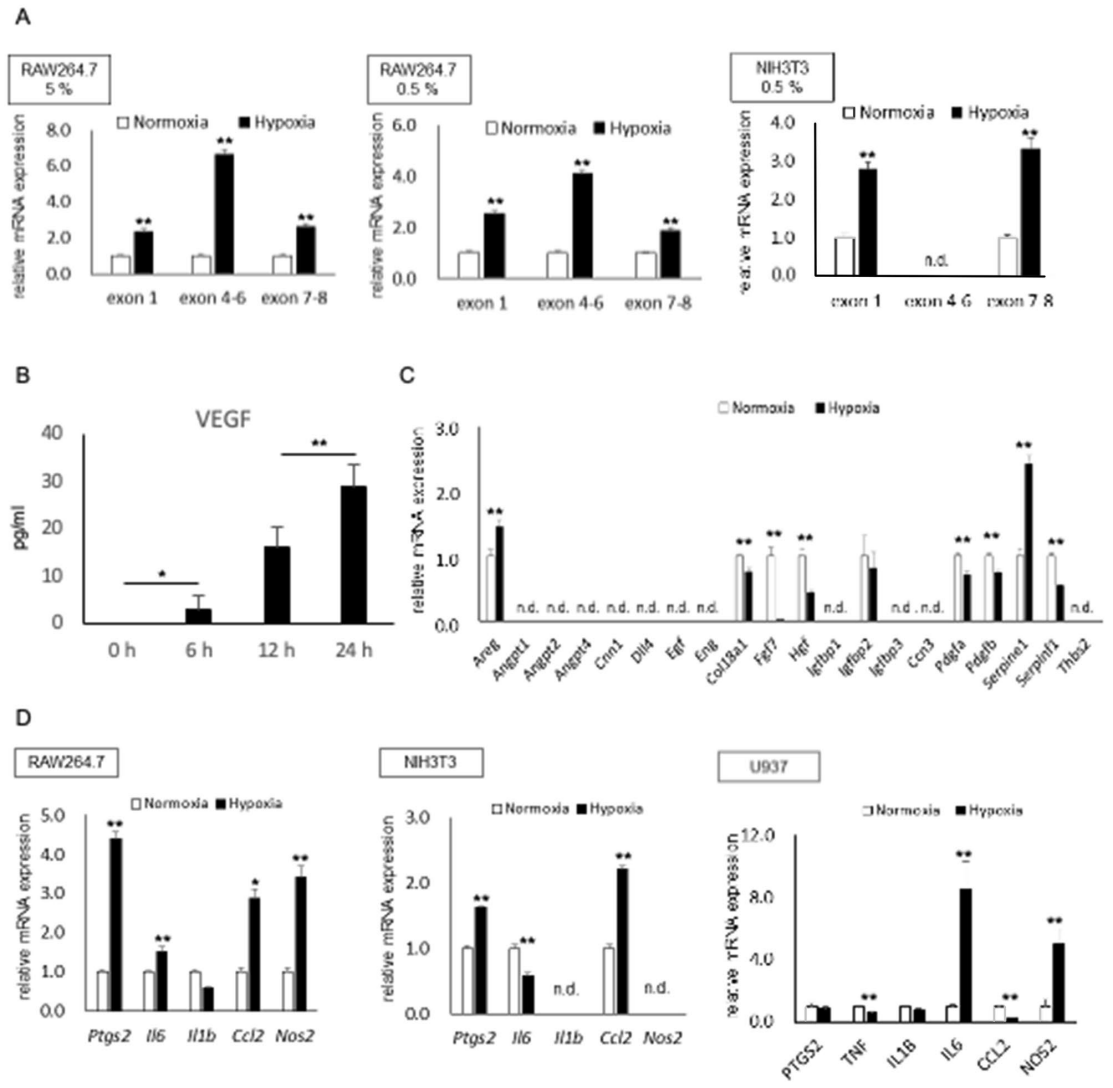
Induction of VEGF (**A,B**), angiogenic factors (**C**) or pro-inflammatory factors (**D**) in cultured cells in vitro. RAW264.7 cells, NIH3T3 cells or U937 cells were cultured in hypoxic condition (5% or 0.5%) for 8 h (**A,C,D**) or indicated time period (**B**). The induction of *Vegf* (**A**), angiogenic factors (**C**) or pro-inflammatory factors (**D**) was then examined by RT-PCR analyses (**A,C,D**, n = 6) and the secretion of VEGF by ELISA (**B**, n = 6). Data represents as Mean and S.E.M. Statistical analysis was done by a non-parametric Mann–Whitney test or by a Kruskal–Wallis test followed by a Steel test. *p < 0.05, **p < 0.01.

As intracranial arteries histologically lack vasa vasorum^22^, the adventitia of intracranial arteries consists of the hypoxic microenvironment, approximate 40 mmHg^23–25^ which corresponds to 5% oxygen. We thereby first examined the effect of the physiological hypoxic condition at the adventitia (5% oxygen) on the expression of a major angiogenic factor, VEGF, in cultured macrophages, RAW264.7 cells. We then found that hypoxia at the physiological level in the adventitia induced expression of *Vegf* in RAW264.7 cells (Fig. 4A, Supplementary Table S2). We next loaded cells on much lower oxygen partial pressure (0.5%), the pathological hypoxia which may be loaded in inflammatory microenvironment of the lesions where many inflammatory cells accumulated and many types of cells activated. Such a pathological hypoxic condition also induced the expression of *Vegf* as well not only in cultured macrophages, RAW264.7 cells, but also in cultured fibroblasts, NIH3T3 cells (Fig. 4A, Supplementary Table S2). In cultured fibroblasts, the alternative splicing form of VEGF lacking exon 6 might be produced. The secretion of VEGF protein from hypoxia-treated RAW264.7 cells was also confirmed by ELISA (Fig. 4B). The expression of angiogenesis-related factors other than *Vegf* in hypoxia-stimulated RAW264.7 cells was also examined. Only a small number of factors was induced under the hypoxic condition, indicating the central role of VEGF in macrophages under hypoxic condition (Fig. 4C, Supplementary Table S2).

In addition, hypoxia loaded on cells induced expressions of some pro-inflammatory genes in various types of cells including *Ptgs2* (a gene for COX-2) or *Ccl2* (Fig. 4D, Supplementary Table S2). The induced expression of COX-2 might exacerbate inflammation via forming the positive feedback loop, COX-2-Prostaglandin E_2_-Prostaglandin E receptor subtype 2 (EP2)-NF-κB^3^, and the secretion of Ccl2 forms the auto-amplification of macrophages^3^, indicating the further amplification of chronic inflammation in situ.

### The expression of VEGF in IA walls induced in rats

**Figure 5:**
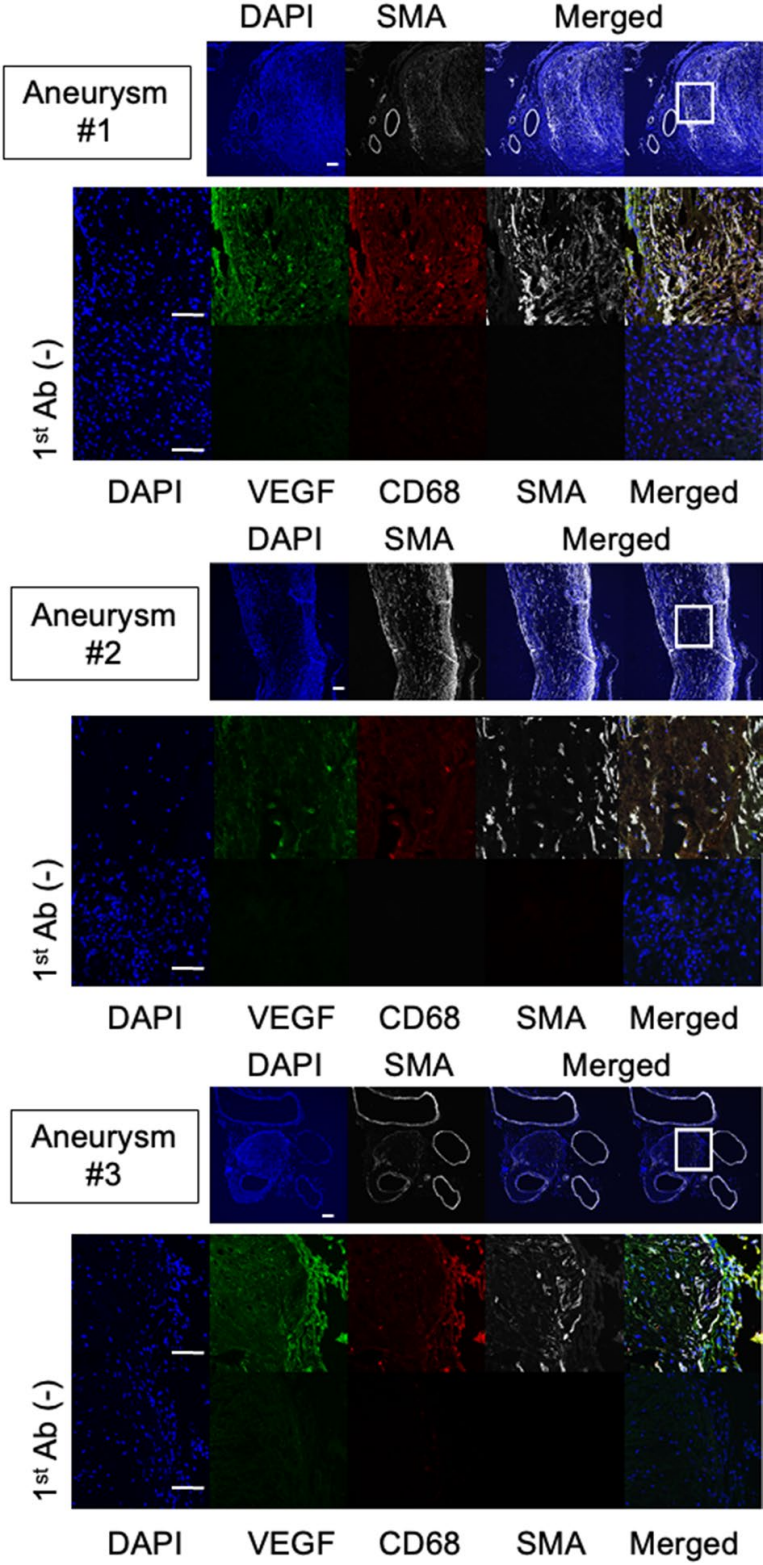
Expression of VEGF in rupture-prone intracranial aneurysm (IA) lesions induced in rats. The images of immunofluorescent staining of IA lesions for VEGF (green), the marker for macrophages, CD68 (red), the marker for smooth muscle cell, α-smooth muscle actin (SMA, gray), nuclear staining by DAPI (blue), and merged images are shown. The images from immunohistochemistry without a primary antibody were served as a negative control. Magnified images corresponding to the square in the upper panels are shown in the lower panels. Scale bar: 50 μm.

We next examined whether VEGF was indeed expressed in rupture-prone IAs, where vasa vasorum was induced^18^, in immunohistochemistry. The signals for VEGF were then detected in IA lesions in immunohistochemistry. Because the most signals for VEGF were well co-localized with those for the macrophage marker, CD68, the major source to produce VEGF in rupture-prone IA lesions was macrophages infiltrating in the lesions (Fig. 5). The induction of VEGF from infiltrating macrophages was supported in above in vitro experiment.

### The expression of VEGF in human IA walls

**Figure 6:**
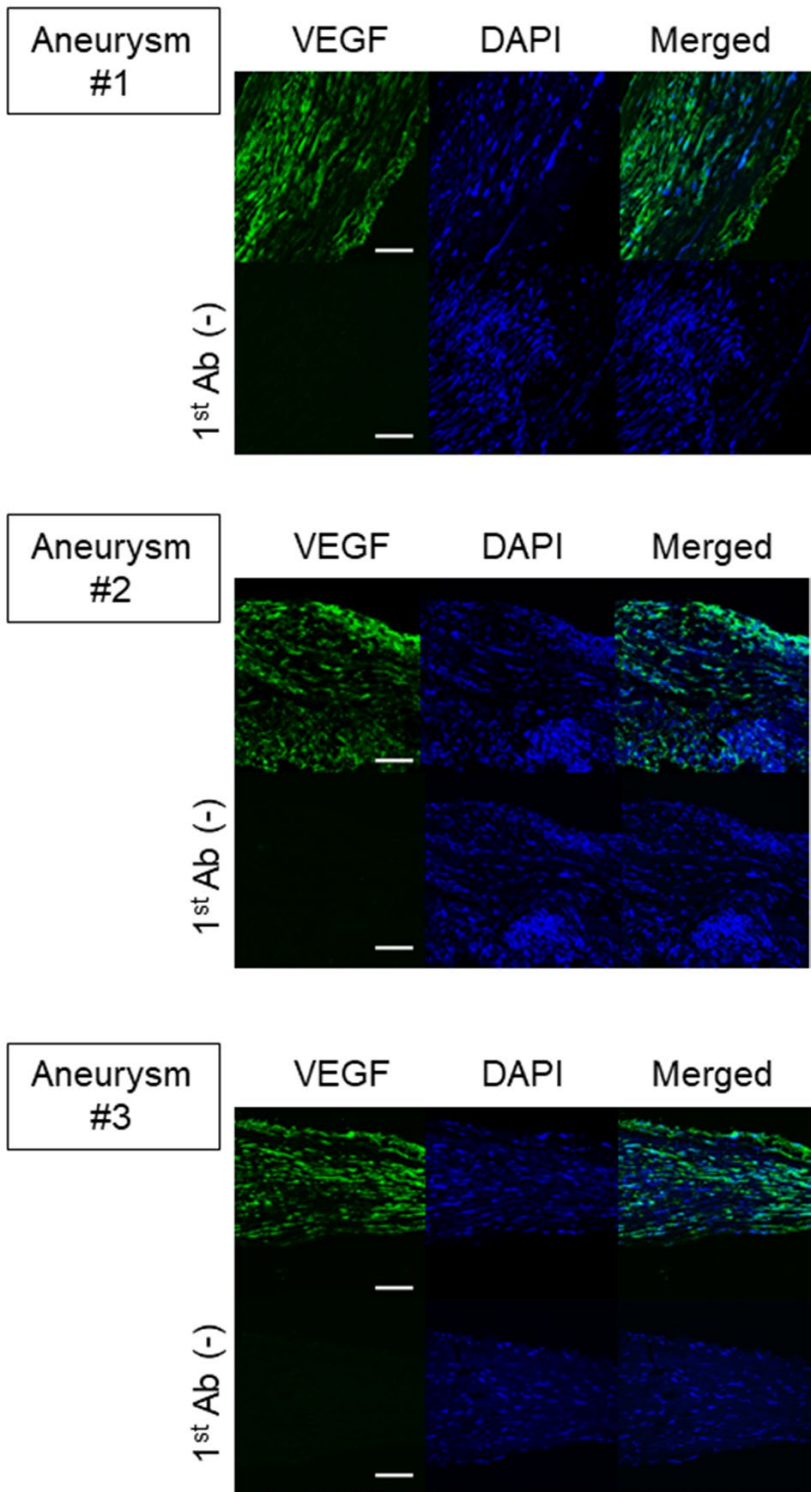
Expression of VEGF in human unruptured intracranial aneurysm (IA) lesions. The images of immunofluorescent staining of IA lesions for VEGF (green), nuclear staining by DAPI (blue), and merged images are shown. The images from immunohistochemistry without a primary antibody were served as a negative control. Scale bar: 50 μm.

To evaluate the clinical relevance of the results from animal experiments, we immunostained slices from human IA lesions to examine the expression of VEGF. The signals for VEGF in immunohistochemistry could be observed and be widely distributed in all the specimens from human cases with unruptured IAs (Fig. 6), suggesting the sustained production of VEGF to some extent during IA progression.

The above results combined together might indicate the accumulation of VEGF from infiltrating macrophages in addition to sustained production during the process leading to rupture of IAs. The results that in human IA lesions VEGF constitutively expressed might, furthermore, indicate the necessity of the close of the dome of IAs to the arteries on brain surface and the collapse of CSF space and the disruption of arachnoid which could be physical barrier to prevent the migration of neovessels.

### The VEGF-dependent induction of vasa vasorum from arteries on brain surface

**Figure 7:**
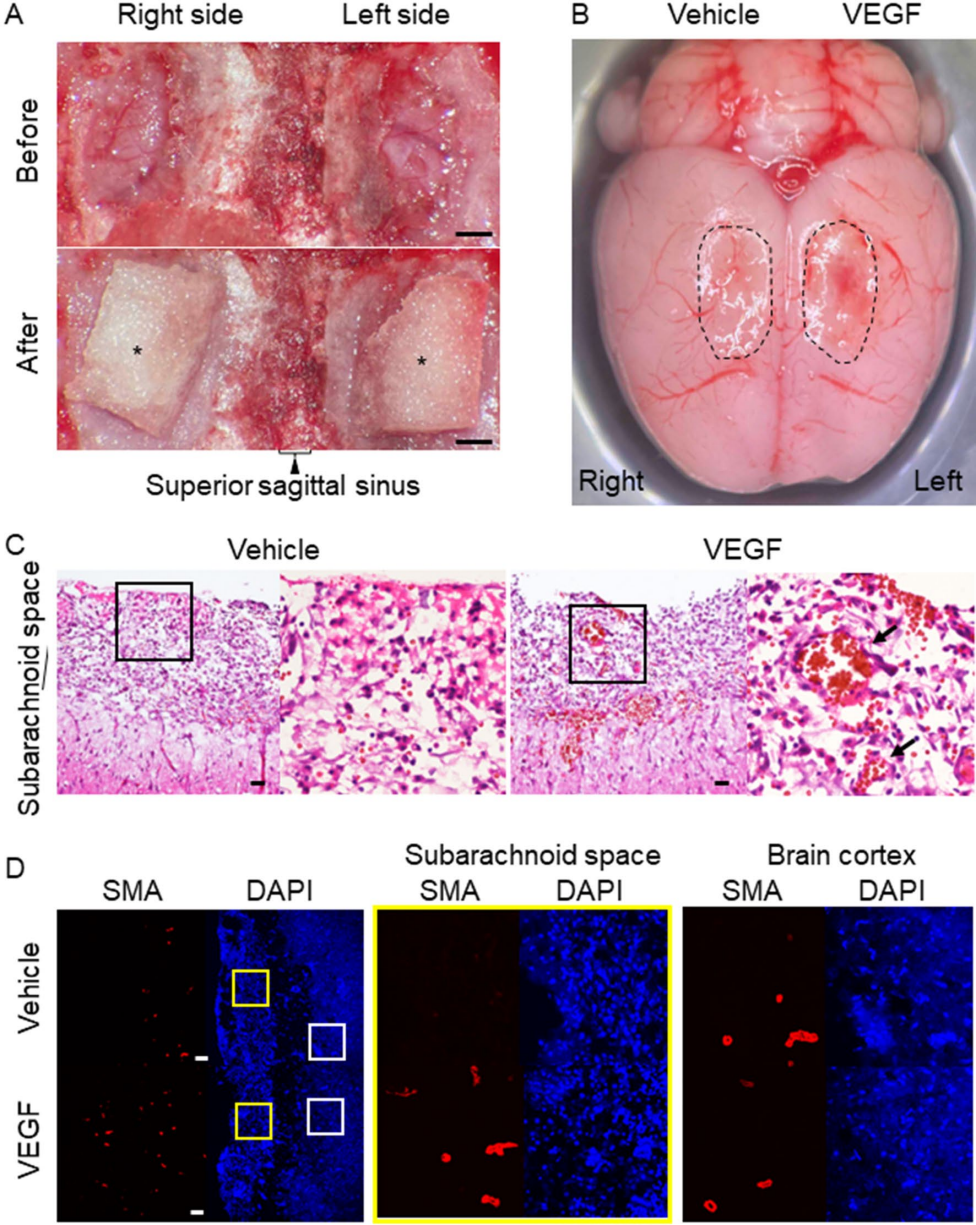
VEGF-mediated induction of neovessels in subarachnoid space. The sheet for slow-release of VEGF or vehicle was placed on the right or the left of brain surface in the same rat (**A**) and the induction of vasa vasorum in subarachnoid space was examined. Asterisks indicate the sheets placed on brain surface. Scale bar: 1 mm. The macroscopic image of the brain surface (**B**) and the histopathological images from hematoxylin–eosin staining from specimen of vehicle- or VEGF-treated side (**C**) are shown. Dotted circles in (**B**) indicate the region where the sheet was placed. Noted the reddish appearance only in the VEGF-treated side indicating the induction of neovessels. Magnified images corresponding to the square in the left panels in (**C**) are shown on the right. Arrows indicate the neovessels induced in subarachnoid space. Scale bar: 50 μm. The images of immunofluorescent staining for the marker for smooth muscle cell, α-smooth muscle actin (SMA, red), nuclear staining by DAPI (blue), and merged images are shown in (**D**). Magnified images corresponding to the squares in the left panels are shown on the right. Scale bar: 100 μm.

To corroborate the induction of vasa vasorum from arteries on brain surface by VEGF, the effect of recombinant VEGF slowly-released into the subarachnoid space on the migration of neovessels was examined in vivo. The sheet for slow-release containing recombinant VEGF or vehicle was placed on brain surface (Fig. 7A) and the induction of neovessels in the subarachnoid space was histologically assessed. As a result, the presence of small arteries with medial smooth muscle cell layer could be observed only in the subarachnoid space of the VEGF-treated side (Fig. 7B–D, Supplementary Fig. S1), confirming the VEGF-dependent neovessel formation from arteries on brain surface.

## Discussion

Considered with the devastating outcome once after the onset of SAH by the rupture of IAs^1,2^ and the nature as an asymptomatic lesion before rupture, machineries underlying the rupture can be a major therapeutic target. Through our previous studies^10,18^, the formation of vasa vasorum at the adventitia, which provides the root for inflammatory cells to infiltrate in the lesions, is presumably the crucial step to drive IAs toward rupture. Thereby, the mechanism promoting the formation of vasa vasorum could be a therapeutic target. Because one of the most potent inducers for neovascularization is hypoxia and hypoxia-induced VEGF functions as a crucial mediator in this step^19–21^, we in the present study examined the potential contribution of hypoxia and hypoxia-induced VEGF expression in IA lesions. We have then clarified the expression of VEGF at the prospective site where IA ruptures. Experiments using Hypoxyprobe have revealed the hypoxic microenvironment at the adventitia of intracranial arteries. Considered with the potent induction of VEGF among angiogenesis-related factors mainly from macrophages, the accumulation of this type of cells may increase the concentration of angiogenic factors in situ to induce the migration of intracranial arteries leading to the formation of vasa vasorum. Because VEGF can also increase permeability of endothelial barrier^28–30^, the increase in VEGF in situ may further facilitate the accumulation of macrophages exacerbating the macrophage-mediated inflammation. Here, VEGF expression is transcriptionally induced by the activation of NF-κB^31–34^ and we have clarified the activation of this transcription factor in macrophages as a major regulator of inflammatory responses in lesions^3,6^. Thereby, macrophage-mediated inflammatory environment in situ presumably further accelerates the secretion of VEGF from macrophages and accumulation of this angiogenic factor in tissues. In addition to the triggering or the exacerbation of inflammatory responses, macrophages again plays the crucial role in the formation of vasa vasorum through VEGF production. Intriguingly, hypoxia induces the expression of pro-inflammatory genes which significantly contribute to the pathogenesis of IAs^3,4,8,9,12,35–38^. Among these factors, COX-2 forms the positive feedback loop consisting of COX-2-Prostaglandin E_2_-EP2-NF-κB, which we have identified as a crucial one to regulate chronic inflammation in lesions^3,38^, and CCL2 forms the auto-amplification loop among macrophages to provide sites for macrophage-mediated chronic inflammation to maintain inflammatory responses^3,4,38^. The induction of COX-2 and CCL2 therefore results in the exacerbation of inflammatory responses presumably leading to excessive degeneration of arterial walls and to rupture of the lesions. These results combined together suggest the multiple roles of hypoxia, one is to provide the root for inflammatory cells to effectively infiltrate via the formation of vasa vasorum and the other is the exacerbation of inflammation, in the process triggering rupture of IAs. In this sense, chronic inflammatory responses still functions as a crucial machinery regulating the disease in addition to the role in the initiation and growth of the lesions^3,6,12^. Such a concept is in line with the clinical evidence that drugs with anti-inflammatory effect like statins or aspirins exert the suppressive effect on the onset of SAH in the case–control studies^39–41^.

In the present study, we have observed the origin of vasa vasorum as arteries attached to brain surface. Intracranial arteries, where IAs are formed, pass through the subarachnoid space and are anchored by arachnoid in places in CSF space. The adventitia in intracranial arteries thus primarily contact with CSF. Not only the distance between the adventitia of the lesions and arteries on brain surface but also CSF space therefore become the physical barrier to prevent the elongation of neovessels to the adventitia from the brain surface. The close of the dome of IAs to the arteries at brain surface and/or the collapse of CSF space might be a necessary factor to the formation of vasa vasorum, leading to rupture of the lesions. The sustained expression of VEGF in unruptured IA lesions presumably being in the hypoxic condition in nature might support the above hypothesis. Here, it is well known that the larger size or the specific location of IAs significantly increases the risk of rupture of IA lesions^42,43^. These two well-established risk factor can narrow the space to facilitate the formation of vasa vasorum at the adventitia.

Recently, the potential of vessel wall imaging by contrast-MRI as a diagnostic method to stratify dangerous IA lesions from many stable ones has been indicated^44^. The precise mechanism of the enhancement in danger lesions, ruptured or growing ones, has not been revealed until now. However, because the arterial wall in vasa vasorum is often leaky and also the blood flow is slow, the enhancement in this imaging method may reflect the presence of vasa vasorum which we have identified as the potential machinery to trigger rupture of IAs.

The major limitation of the present study is the lack of experimental evidence that VEGF in IA lesions indeed plays a crucial role to the rupture of IAs through inducing vasa vasorum formation at the adventitia. For example, the genetic deletion of VEGF in rats or the intrathecal administration of anti-VEGF antibody to neutralize VEGF from macrophages may become potential experiments. However, both experiments are challenging. We have used the rat model of IAs in a series of studies and the establishment of a rat line deficient in *Vegf* specifically in macrophages, which is necessary for experiments because the genetic deletion of VEGF in a whole body is lethal^45^, is technically difficult. Furthermore, it takes long, several months, to assess the effect of a drug or an antibody on the onset of SAH in rats, making the repeat intrathecal administration of anti-VEGF antibody being necessary.

## Conclusions

Considered with the poor outcome of SAH due to rupture of IAs, the development of a novel therapeutic strategy to prevent rupture based on the pathogenesis is mandatory for social health. Based on the recent experiment findings that vasa vasorum formation is the potential machinery to trigger rupture of IAs via facilitating the infiltration of inflammatory cells to exacerbate chronic inflammation in situ, we in the present study examined mechanisms regulating the formation of vasa vasorum. We then found the presence of hypoxic microenvironment at the adventitia of intracranial arteries where IA is induced. Among angiogenic factors, the production of VEGF in the lesions including infiltrating macrophages and the potential contribution of this angiogenic factor to the induction of vasa vasorum in vivo were revealed. Here, because intracranial arteries histologically lack vasa vasorum and are surrounded by CSF space, the formation of the hypoxic microenvironment could be a character of intracranial arteries and inflammatory responses there might further facilitate such a condition to induce vasa vasorum. In addition, the enlargement of the lesions and the disruption of physical barrier like arachnoid could lead to the migration of vasa vasorum from arteries at brain surface. The findings from the present study have thus revealed the potential role of hypoxic microenvironment and hypoxia-induced VEGF production as a machinery triggering the rupture of IAs via providing root for inflammatory cells to exacerbate inflammation in the microenvironment of the lesions.

## Supplementary Information

Supplementary Figure S1.

Supplementary Legends.

Supplementary Table S1.

Supplementary Table S2.

## Data Availability

The datasets used and/or analyzed during the current study are available from the corresponding author on reasonable request.

## Abbreviations

COX-2: Cyclooxygenase-2
CSF: Cerebrospinal fluid
EP2: Prostaglandin E receptor subtype 2
IA: Intracranial aneurysm
RT-PCR: Real time-PCR
SAH: Subarachnoid hemorrhage
SMA: α-Smooth muscle actin
VEGF: Vascular endothelial growth factor
