# Supplementary figures and images for: Hypoxic microenvironment as a crucial factor triggering events leading to rupture of intracranial aneurysm
Source: Sci Rep. 2023 Apr 4;13:5545. doi: 10.1038/s41598-023-32001-z (PMC10073088; doi:10.1038/s41598-023-32001-z)

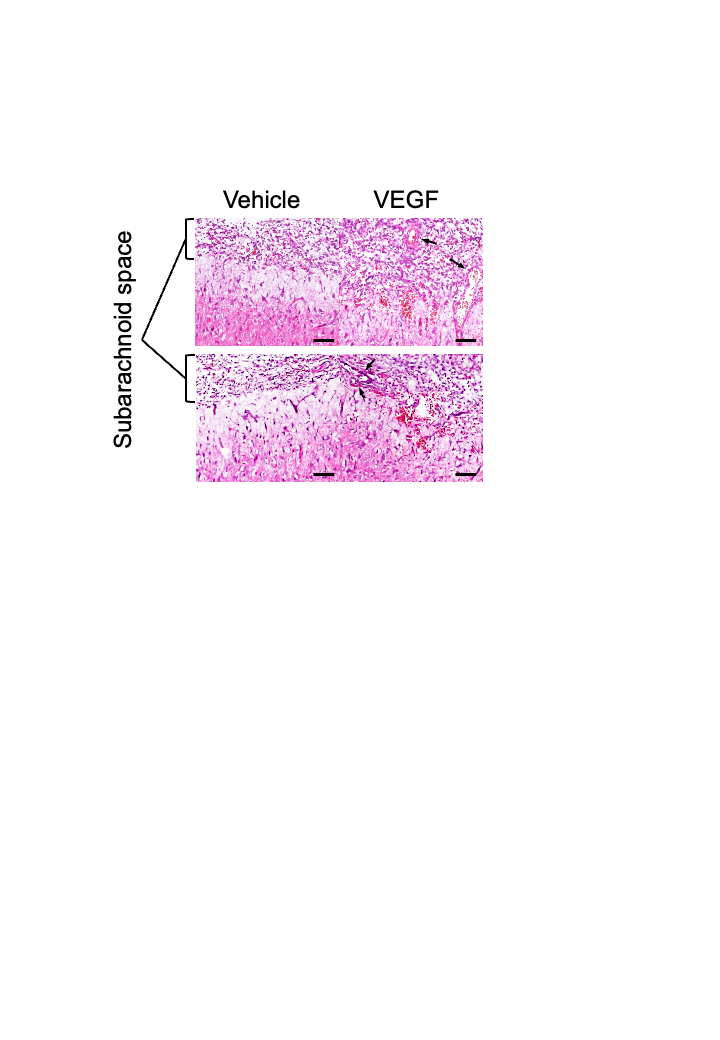

Supplement: Supplementary file 1 — Supplementary Figure S1. [file 41598_2023_32001_MOESM1_ESM.tiff]
