# Supplementary material for: Hypoxic microenvironment as a crucial factor triggering events leading to rupture of intracranial aneurysm
Source: Sci Rep. 2023 Apr 4;13:5545. doi: 10.1038/s41598-023-32001-z (PMC10073088; doi:10.1038/s41598-023-32001-z)
Supplement: Supplementary file 2 — Supplementary Legends. [file 41598_2023_32001_MOESM2_ESM.docx]

**Supplementary Fig. S1. VEGF-mediated induction of neovessels in subarachnoid space**

The sheet for slow-release of VEGF or vehicle was placed on the right or the left of brain surface in the same rat as in Fig. 7 and the induction of vasa vasorum in subarachnoid space was examined. The histopathological images from Hematoxylin-Eosin staining from specimen of vehicle- or VEGF-treated side in two rats other than shown in Fig. 7 are shown. Arrows indicate the neovessels induced in subarachnoid space. Scale bar: 100 μm.
